# Perioperative Diagnosis of Acute Pulmonary Embolism Following Laparoscopic Hysterectomy Under General Anaesthesia: A Rare Case Report

**DOI:** 10.4274/TJAR.2025.252004

**Published:** 2026-02-09

**Authors:** Thang Phan, Lanh Tran Thi Thu, Trong Binh Le, Braydon Bak, Minh Nguyen Van

**Affiliations:** 1 Hue University, University of Medicine and Pharmacy, Hue City, Vietnam; 2 Mayo Clinic Hospital, Clinic of Anaesthesiology and Perioperative Medicine, Minnesota, United States of America

**Keywords:** Laparoscopic hysterectomy, multidisciplinary approach, perioperative care, pulmonary embolism

## Abstract

Perioperative pulmonary embolism (PE) is rare but potentially fatal and often difficult to diagnose under general anaesthesia. A fifty-one-year-old woman with hypertension and type II diabetes underwent laparoscopic hysterectomy. After pneumoperitoneum and Trendelenburg positioning, she developed hypoxemia, decreased EtCO₂, and hypotension. Hemodynamics improved after de-sufflation, but hypoxemia persisted post-extubation. Echocardiogram showed right heart strain, and computed tomography pulmonary angiography confirmed acute PE from lower extremity deep vein thrombosis. She was treated with anticoagulation therapy, vasopressor support, and inferior vena cava filter placement and discharged from intensive care unit on postoperative day 5. This case highlights the importance of early suspicion and prompt diagnostic evaluation of intraoperative PE. A multidisciplinary approach and timely anticoagulation with or without interventional therapy are critical to improve outcomes.

**Main Points**

• Suspect pulmonary embolism with sudden hypoxemia and decreased EtCO₂ after pneumoperitoneum and Trendelenburg positioning in high-risk patients.

• Early echocardiogram and computed tomography pulmonary angiography are key to rapid diagnosis and guiding treatment.

• Early detection perioperative acute pulmonary embolism and a multidisciplinary approach improves survival.

## Introduction

Perioperative acute pulmonary embolism (PE) is relatively rare, but potentially fatal. The development of PE during surgery under general anaesthesia is difficult to recognize, leading to a delay in diagnosis and subsequent treatment. We describe a case of suspected unstable PE diagnosed by clinical characteristics, transthoracic echocardiography, computed tomography (CT) pulmonary angiography (CTPA), and CT venography of the lower extremities. The patient was treated with anticoagulation therapy and inferior vena cava filter insertion. The patient was discharged from the intensive care unit (ICU) on postoperative day (POD) five. Although laparoscopic surgery-related PE is uncommon, early detection and a multidisciplinary approach are important in the management of this life-threatening complication to improve patient outcomes.

## Case Report

Written consent has been obtained from the patient indicating his approval for publication. A fifty-one-year-old female, body max index 26 kg m^2-1^, with a history of hypertension and newly diagnosed diabetes type II presented with a chief complaint of menorrhagia secondary to uterine adenomyosis. The patient was taking medically prescribed oral contraceptives (Desogestrel) and tranexamic acid, as well as self-prescribed herbal medication (Panax notoginseng), 3 months prior to the operation. She was scheduled for a laparoscopic total hysterectomy under general anaesthesia due to failure of medical management to resolve her symptoms. Preoperative workup included a transthoracic echocardiogram that was unremarkable, with normal biventricular systolic function and a left ventricular ejection fraction of 72%. Chest X-ray was also unremarkable. Baseline vital signs were within normal ranges, including a heart rate (HR) of 88 bpm, blood pressure (BP) 135/80 mmHg, SpO_2_ 99% on room air. Airway and cardiopulmonary examination was normal.

The patient was induced with intravenous fentanyl (2 µg kg^-1^), propofol (3 mg kg^-1^), and rocuronium (0.6 mg kg^-1^). Direct endotracheal intubation was atraumatic, and the patient was maintained under general anaesthesia with sevoflurane. The patient’s abdomen was insufflated with CO_2 _gas to 12 mmHg, was placed in Trendelenburg position, and the surgery progressed uneventfully. Twenty minutes into the case, the SpO_2_ suddenly dropped from 99% to 94%. Vitals were BP 110/70 mmHg, HR 86 bpm, EtCO_2_ 35 mmHg, and Ppeak 22 cmH_2_O. On a clinical exam, the endotracheal tube remained in appropriate position, lung sounds were clear without rales, and the SpO_2_ remained between 94% and 96%. However, after 5 minutes, the SpO_2_ dropped to 84%, and the EtCO_2_ decreased to 32 mmHg with a BP of 120/70 mmHg. Airway pressures remained normal: (Ppeak 22 cmH_2_O), and a expiratory tidal volume (VTe) of 7 mL kg^-1^ was maintained. The surgery team was notified; the abdomen was desufflated and the patient was repositioned to the zero degree supine position. The ventilator was checked, minimal sputum was aspirated from the endotracheal tube, and the patient was ventilated manually with an ambu bag. After 5 minutes, SpO_2_ improved to 97% and BP improved to 115/70 mmHg.

**Figure 1:**
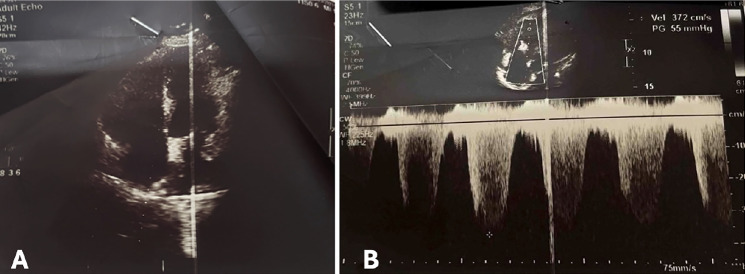
Transthoracic echocardiogram shows right ventricular strain (A) and evidence of pulmonary hypertension with tricuspid regurgitation pressure grafient 55 mmHg (B).

**Figure 2:**
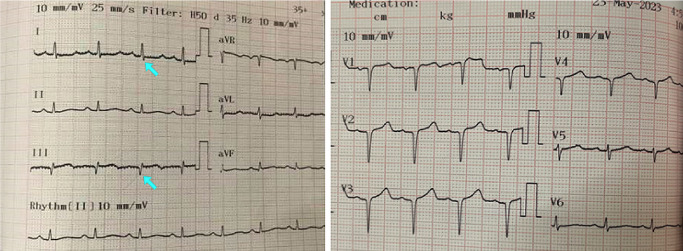
S1Q3 in electrocardiogram.

**Figure 3:**
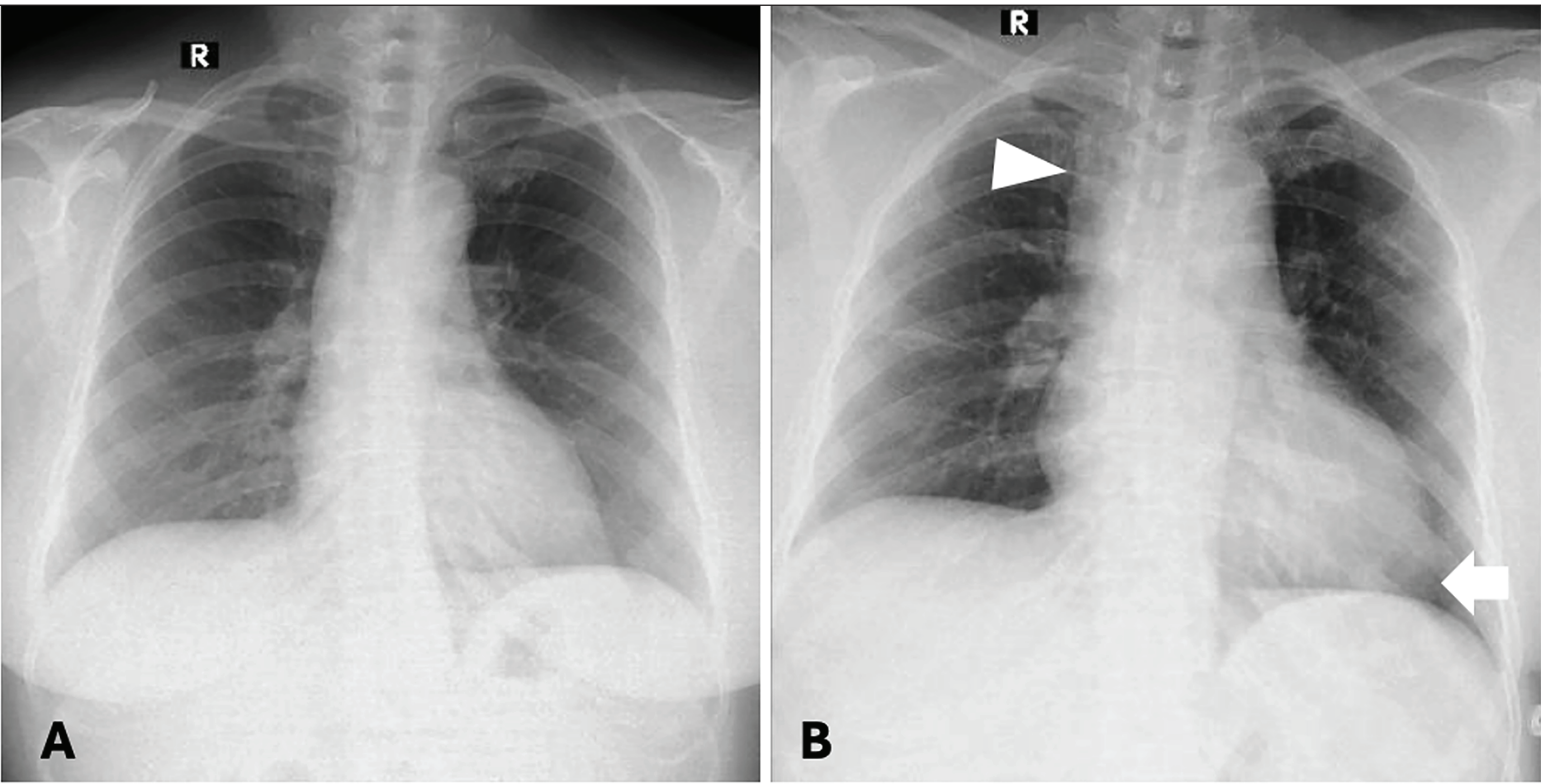
Comparison of chest X-ray before and after operation. Preoperavite chest X-ray (A) was normal. On postoperative X-ray, cardiomegaly with rounded left heart border and uplifted cardiac apex was appreciated, compatible with right ventricle dilatation (arrow). Widening of the right paratracheal stripe was also noted, suggesting an enlargement of the superior vena cava (arrowhead).

**Figure 4:**
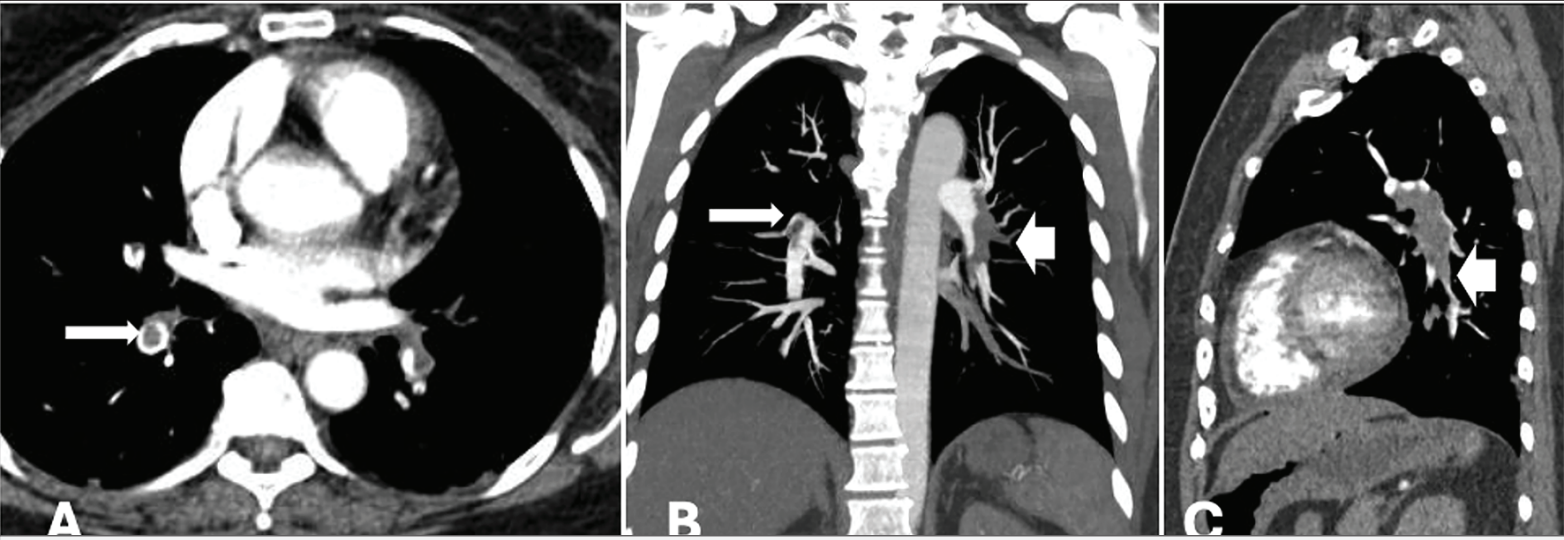
Acute pulmonary embolism was confirmed on computed tomography pulmonary angiogram (CTPA). Axial CTPA image (A) shows rim sign in the right middle lobe pulmonary artery (long arrow), corresponding to a focal thrombi seen on coronal view (B). Massive acute thrombosis of the left pulmonary artery extending to the interlobal artery and the anterior branch (short arrow) was appreciated (B, C).

**Figure 5:**
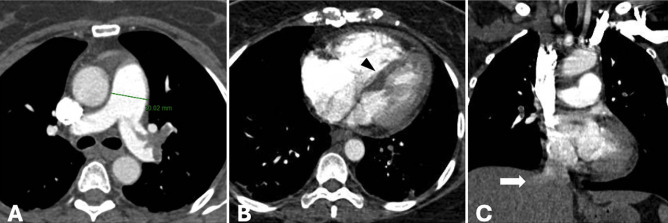
Pulmonary hypertension was suggestive on computed tomography pulmonary angiography with (A) enlargement of the pulmonary artery trunk, (B) dilatation of the right ventricle and flattening of the interventricular septum (arrowhead), and (C) reflux of the contrast media to the right hepatic vein (arrow).

**Figure 6:**
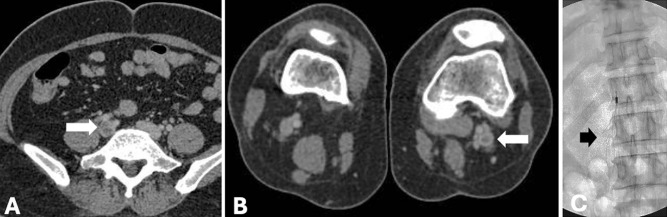
Deep vein thrombosis was seen at (A) the right common iliac vein and (B) the left popliteal vein (arrow) noted the vessel wall thickening and enhancement indicating an acute phase of thrombosis. (C) An inferior vena cava filter was inserted (short arrow).

At this point, it was decided to resume the procedure laparoscopically. However, upon abdominal insufflation and positioning the patient in Trendelenburg, the SpO_2_ dropped rapidly to 89-90%, EtCO_2_ decreased from 38 mmHg to 30 mmHg, and BP dropped to 95/50 mmHg. Due to the hemodynamic changes with insufflation, the surgical team decided to convert to open surgery. Following abdominal desufflation and opening of the abdomen, the patient’s vitals remained stable with an SpO_2_ of 97-98%, HR of 86-95 bpm, BP of 110/80-130/80 mmHg, and EtCO_2_ of 32-35 mmHg. The patient was extubated without difficulty when the procedure was completed 1 hour later. However, shortly after extubation, the SpO_2_ decreased to 90-92% on room air, and an arterial blood gas (ABG) was indicated. Supplemental O_2_ was administered via nasal cannula at 5 L min^-1^ and she was transferred to the recovery room with SpO_2_ 94% and BP 105/60 mmHg. After excluding possible surgical causes, we had a high suspicion of PE due to air or thrombi, with an simplified Pulmonary Embolism Severity Index score of 3 points. A transthoracic echocardiogram was performed showing right heart dilatation, mild tricuspid regurgitation, and pulmonary artery systolic pressure of 60 mmHg (Figure 1). The electrocardiogram showed sinus rhythm and S1Q3 sign (Figure 2). A chest X-ray showed evidence of cardiomegaly (Figure 3). The remarkable laboratory tests showed elevated D-dimer 4213 ng mL^-1^, lactate 2.1 mmol L^-1^, hs-troponin T 0.021 ng mL^-1^, Pro BNP 1601 pg mL^-1^, and ABG results with pH 7.36, PaO_2_ 90 mmHg, PaCO_2_ 35 mmHg. Acute PE was strongly suspected. A CTPA was obtained to confirm the presence of a PE (Figure 4), which also showed evidence of pulmonary hypertension (Figure 5). Deep vein thrombosis was also evident under ultrasound and CTPA (Figure 6).

Multidisciplinary consultations, including cardiology, interventional radiology, obstetrics and gynecology, cardiothoracic surgery, and critical care, confirmed acute PE secondary to deep vein thrombosis. The patient was treated with intravenous heparin bolus 5000 IU followed by a 500 IU/hour infusion. The patient was admitted to the ICU for hemodynamic monitoring requiring a noradrenaline infusion up to 0.1 mcg kg^-1^ min^-1^ for 24 hours. Deep venous thrombosis ultrasound and CT venography of the lower extremities were performed, showing the left popliteal vein and acute thrombosis localized to the right common iliac vein, respectively. An inferior vena cava filter was subsequently inserted by interventional radiology via a transjugular approach (Figure 6). On POD 3, the heparin infusion was discontinued, and the patient was transitioned to oral rivaroxaban, 30 mg day^-1^. The patient was discharged from the ICU on POD 5.

## Discussion

Unstable acute PE is a medical emergency with a high mortality rate, reaching up to 30% if untreated.^1^ Risk factors for PE include conditions that impair venous elasticity, vascular disorders that damage or disrupt endothelial function, and a hypercoagulable state.^2^ Non-specific symptoms of PE may include dyspnea, pleuritic chest pain, headache, fatigue, syncope, and may lead to cardiac arrest.^3, 4^ However, diagnosing PE in patients under general anaesthesia may be challenging.^5^ The incidence of PE during surgical procedures is a rare complication associated with a perioperative mortality rate of approximately 12.5%.^6^

In this case, the female patient presented with two groups of risk factors:

**1. Factors Promoting Thrombus Formation:** The patient had a history of cardiovascular disease (hypertension and diabetes) and was taking oral contraception, tranexamic acid, and Panax notoginseng for the treatment of menorrhagia. Some traditional herbs commonly used in Vietnam to treat menorrhagia include Panax notoginseng, Typha angustifolia, Eclipta prostrata, Styphnolobium japonicum, and Glycyrrhiza uralensis.^7^ Panax notoginseng contains active compounds, including dencichin and flavonoids, that promote blood coagulation in the body.^8^ Using tranexamic acid and Panax notoginseng concomitantly is a high-risk factor for acute deep vein thrombosis.

**2. Factors Promoting Thrombus Displacement:** Laparoscopic pneumoperitoneum and the Trendelenburg position may enhance thrombus movement from the lower extremities to other organs, specifically pulmonary circulation. The combination of elevated intra-abdominal pressure during laparoscopic surgery and reverse Trendelenburg positioning increases the risk of venous thromboembolism by the combination of elevated intra-abdominal pressure during laparoscopic surgery and reverse Trendelenburg positioning because of their additive effects on venous stasis and impaired venous return. Current evidence suggests that laparoscopic surgery is associated with a reduced risk of postoperative PE compared to open surgical approaches. This observed benefit is largely attributed to enhanced postoperative recovery, including pain control and earlier mobilization, both of which are critical factors in mitigating venous thromboembolic risk.^9^

During the laparoscopic hysterectomy, the patient suddenly experienced a decrease in SpO_2_, EtCO_2_, and hypotension following the establishment of pneumoperitoneum and positioning. Potential causes for these changes could include bronchospasm, pulmonary edema, pneumothorax, cardiogenic shock, hypovolemic shock, or obstructive shock. However, the patient exhibited no evidence of elevated airway pressures; the endotracheal tube was correctly positioned, VTe was adequate, and there was minimal blood loss during surgery. Given the identified patient risk factors, we raised suspicion for PE. Since open hysterectomy has less respiratory and hemodynamic effects compared to laparoscopy, the decision was made to convert to an open procedure after multidisciplinary discussion. According to the European Society of Cardiology guidelines, bedside echocardiography and if feasible, CTPA, should be performed promptly when PE is suspected in a patient with hemodynamic instability.^3^ In this case, the patient, with a ten-year history of hypertension, was suspected of having hemodynamically unstable PE due to hypotension requiring vasopressor support, despite ruling out iatrogenic causes, hemorrhagic and distributive shock. Following the procedure, a CTPA, D-dimer, transthoracic echocardiography, assessment of right ventricular function, and ABG analysis were obtained to establish an early diagnosis of PE and guide management.

The CTPA results confirmed a diagnosis of PE likely secondary to lower extremity DVT. Currently, the American Heart Association and the American College of Chest Physicians recommend systemic thrombolysis as first-line therapy for patients with massive PE and suggest considering thrombolysis for patients with submassive PE. For high-risk patients with absolute contraindications to thrombolytic therapy, catheter-directed therapy, or surgical thrombectomy is recommended.^10, 11^ The patient was admitted to the ICU presenting with mild hypoxemia, HR 115 bpm, BP 95/60 mmHg no altered mental status, and no shock symptoms. Post-hysterectomy, the risk of postoperative bleeding was classified as moderate, placing her in a group contraindicated for systemic thrombolytic therapy. This necessitated careful consideration of the benefits and risks associated with anticoagulation therapy.

Following a multidisciplinary consultation, including cardiology, interventional radiology, obstetrics and gynecology, cardiothoracic surgery, anaesthesia, and intensivists, the patient was started on unfractionated heparin with close hemodynamic monitoring. If hemodynamic instability occurred, intravascular thrombectomy and placement of an IVC filter in the catheterization lab would be considered. According to recommendations, the choice of intervention in cases of PE should be individualized and depend on the available resources.^5^ Invasive methods such as surgical intervention, catheter-directed thrombectomy, or percutaneous thrombectomy; as well as less invasive options thrombolysis or anticoagulation therapy, may be considered. For this clinical case, important considerations in management included a thorough assessment of the patient’s tissue hypoxia status, selection of less invasive treatment, close monitoring of treatment response, and contingency planning for potential complications. Minimally invasive interventions to achieve maximum efficacy are a current trend in treatment.

This patient presented with multiple risk factors for PE including a history of diabetes, oral contraceptive use, tranexamic acid, Panax notoginseng use, and prolonged immobilization post-surgery. Given the significant acute thrombosis in the common iliac and popliteal veins, the placement of an IVC filter was indicated to prevent further progression, following the recommendations of the Society of Interventional Radiology Clinical Practice Guideline.^12^

## Conclusion

Unstable intraoperative PE can be difficult to diagnose in patients under general anaesthesia. PE can be suspected based on the presence of sudden hypoxia, decreased EtCO_2_, and hemodynamic instability after CO_2_ inflation and Trendelenburg positioning, in patients with known PE risk factors. The clinical outcome of our patients who experience intraoperative PE depends on timely diagnosis, multi-specialty coordination, minimally invasive treatment, and interventional resources of the hospital facility.

## Ethics

**Informed Consent:** Written consent has been obtained from the patient indicating his approval for publication.
